# Teaching, learning and assessment methods for sustainability education on the land–sea interface

**DOI:** 10.1007/s43621-023-00120-2

**Published:** 2023-01-19

**Authors:** Andreas C. Bryhn, Andrea Belgrano

**Affiliations:** 1 Department of Aquatic Resources, Institute of Coastal Research, Swedish University of Agricultural Sciences, Skolgatan 6, 74242 Öregrund, Sweden; 2 Department of Aquatic Resources, Institute of Marine Research, Swedish University of Agricultural Sciences, Turistgatan 5, 45330 Lysekil, Sweden; 3 Swedish Institute for the Marine Environment (SIME), University of Gothenburg, Seminariegatan 1F, 41313 Gothenburg, Sweden

**Keywords:** Land–sea interface, Sustainability, Teaching and learning methods, Learner-centered

## Abstract

The Land–Sea Interface (LSI) is where land and sea meet, not only in physical terms, but also with regards to a large variety of ecological and societal aspects. The United Nations has proclaimed the period 2021–2030 the Ocean Decade, which entails striving for a sustainable use of the ocean and teaching and learning about ocean related issues. Teaching and learning about the LSI are also tightly connected with several Sustainable Development Goals (Global Goals) such as Life Below Water, Zero Hunger and Sustainable Cities and Communities. Teaching and learning about sustainability lacks a uniform pedagogy, and it is probably wise to maintain that apparently adaptive diversity. In this globally relevant methods overview, we present a wide range of relatively new and/or successful and mostly largely learner-centered methods. We also discuss how effective and popular they are, and give examples on how most of these methods are already used in LSI sustainability teaching. There will probably not be any successful “one size fits all” model developing for LSI teaching and learning, and each module, course and programme will have to develop its own recipe for successful teaching and learning, possibly with support from one or several methods discussed in this paper.

## Introduction

Variants of traditional higher education has existed for millennia in many parts of the world [1–4]. Traditional higher education is commonly described as more teacher-centered than learner-centered; i.e., mainly consisting of students listening, observing and repeating what a scholar says and does, with teaching and learning typically set in a lecture hall, classroom, or similar [5–7]. Modern higher education often builds upon such traditions, but also involves various degrees of more learner-centered practices where learners have a higher responsibility in acquiring knowledge and skills in interacting, writing, listening, visualising and idea shaping. Although learner-centered education also has ancient roots [8], the range of newer methods involving more learner-centered activities is wide, and widening [5–7, 9]. In recent decades and in many countries, constructive alignment teaching and learning [10, 11] has become mainstream in higher education [12, 13], although it has also been subject to criticism [14]. Constructive alignment is outcome-based, puts the learner activities and active learner participation in the center, and stresses continuous alignment between course objectives and the assessment of student performance. There are many different teaching and learning methods and techniques available, which may be combined with constructive alignment [10, 11, 13].

There is also nowadays an influential paradigm on the global scale in the framework of constructive alignment regarding the use of learning outcomes in higher education [9, 15–17]. Learning outcomes have been defined as “statements of what a learner is expected to know” [9] and may be described as consisting of either or both of two parts: prescribed outcomes and achieved outcomes [18]. Prescribed outcomes are what the student *should* learn during a course or a programme. Achieved outcomes are measurements of what the student *has* learned during a course or a programme [18]. There have been ambitions to shape learning outcomes to become more similar across scientific disciplines, universities and countries, but a large diversity has hitherto remained, providing room for flexibility. There is a vivid academic discussion about the optimal function and shaping of learning outcomes [18, 19]. Outcome should be distinguished from output, which is what the learner produces, and impact, which is the effect or influence on the learner [20].

Another modern feature in higher education is co-creation, which means treating learners like participatory partners in the learning environment, allowing them to contribute to what is learned. Co-creation empowers learners and should be inclusive in order to encourage both highly motivated and less motivated students to become actively engaged [21–23].

The Land–Sea Interface (LSI; sometimes referred to as the coastal transition zone) is where and when land and sea meet; apparently physically, but also in hydrological, ecological, social, economic, cultural, legal, administrative, planning and governance contexts and terms [24, 25]. Studying and addressing problems and challenges at the LSI is a global concern for many reasons, including sustainability, since many of the global Sustainable Development Goals encompass or depend upon the LSI [26]. For instance, the LSI can be sites where rivers discharge anthropogenic pollutants and substances of natural origin, resulting in harmful algal blooms and hypoxia [27]. Coastal ecosystems are home to native and often unique flora and fauna, such as marine mammals, fish, shellfish, seagrass and algae [28, 29]. While being subjected to and affected by numerous natural forces and anthropogenic pressures, these ecosystems also provide a multitude of benefits to humans, so-called ecosystem services [29, 30], as well as general well-being [31]. A substantial proportion (40% according to [32]) of the global human population lives 100 km or less from the sea. People use the LSI for housing, recreation, and for providing for themselves. Coastal and marine tourism is gaining in economic importance [33]. Ports are used for recreational boats, ferries, and marine transportation, the latter being a major and environmentally low-impact way to move goods [34]. Marine spatial planning involves addressing governance and management issues including conflicts of interest in coastal and offshore marine waters [35]. Ecosystem-based management in the coastal zone aims at bringing together all relevant scientific disciplines and stakeholders to solve complex issues in the LSI in a sustainable way [36]. Thus, the LSI has many economic, ecological and social sustainability aspects and involves a large number and a wide range of scientific disciplines.

Sustainability is in itself gradually gaining importance in higher education [37]. The United Nations proclaimed the period 2005–2014 the decade of education for sustainability [38]. It subsequently proclaimed 2021–2030 a decade of ocean science, including aspirations to teach and learn about the ocean (see https://www.oceandecade.org/). Due to the substantial scientific and societal interest for the LSI in particular, in combination with sustainability, sustainability in the LSI has become subject to modules, courses and programmes in higher education in many different parts of the world.

There is a research field of its own called Education for Sustainability or Education for Sustainable Development, aiming to implement sustainability in a wide range of subjects, wherever appropriate [39]. This requires commitment from both faculties and teachers [40]. In Education for Sustainability, a distinction is made between education *on* the environment (focusing on knowledge), education *in* the environment (focusing on emotions) and education *for* the environment (focusing on action) [41]. Each of these three categories needs specifically designed pedagogy, teaching and learning methods, and learning outcomes.

A small selection of recently or currently offered LSI and sustainability related courses and programmes in higher education is provided in Table 1.

**Table 1:** A selected sample of higher education courses and programmes currently (2022) or recently taught, with connections to sustainability in the Land–Sea Interface

Course or programme name	Country
Analysing the interdependencies of land and sea [42] Maritime operations and management [43] Climate change – effects on the landscape and potential solutions [44] Coastal hazards management [45] Biological oceanography and conservation [46] Regional ocean governance [47] Human and ecological systems [48] Ocean macroturbulence and its role in Earth’s climate [49] Marine GIS applications for coastal zone management [50] Coastal and marine science [51] Maritime archaeology and underwater cultural heritage [52] Sea level rise and coastal cities: science, policy and practice [53] Society, environment and sustainability [54] Experimental work in marine ecology [55]	Romania United Kingdom Sweden India Pakistan Thailand Japan China Malaysia Australia Egypt United States Canada Chile

The pedagogy used in sustainability education is not uniform but varies to a substantial degree [19]. In line with allowing for and promoting this adaptive variation, the present study explores the actual and potential use of different teaching and learning methods in programmes and courses relating to sustainability in the LSI. The idea is to elaborate a globally relevant overview of recent literature on successful, popular and/or novel methods with practical examples for improved teaching about sustainability in the LSI as they are being applied in different parts of the world. We will also discuss which novel methods are more or less suitable for teaching sustainability in the LSI, which will hopefully be useful for teachers and learners worldwide.

## Methodology

Literature searches were made in Google Scholar and Web of Science regarding popular and/or novel methods for teaching, learning and assessment. Such searches, in addition to regular browser searches for grey literature and web pages, were also made to determine the extent to which these methods are currently used for sustainability education regarding the LSI. Focus was on the methods rather than on the education programmes. We aimed at identifying examples in different parts of the world to make the overview more relevant in a global context.

## Relevant teaching, learning and assessment methods for sustainability education on the land–sea interface

This section presents popular and/or novel teaching and learning methods, which may be relevant for the LSI, followed by some occasionally used modern assessment methods. These assessment methods in higher education were included in the section since assessment is an essential part of the learning process [56, 57].

### Traditional classroom learning

Traditional classroom learning may be the most common form of learning in higher education. It is the “gold standard” against which other types of learning is usually compared [58–61]. It also tends to obtain comparatively high ratings by university students [5, 58]. The teacher and learners are typically located in a lecture hall or a classroom, where the teacher lectures, and is often supported by some kind of visual aid [6, 61]. It may be a challenge to adapt classroom learning to constructive alignment, since the latter requires active learner participation. For instance, special individual or group tasks building on examples from the real world could improve learner engagement and activity in classroom learning [12]. Another example is to have learners present and defend work that they have done, and if the peer and teacher criticism is constructive, that could benefit active learning [62]. In an LSI setting, traditional classroom learning is applied to acquiring basic knowledge about foodwebs, coastal biodiversity, port design, maritime legislation, coastal cultural features, coastal currents, sediment-water interactions, marine governance, fisheries science, protection against sea-level rise, coastal ecosystem services, and the influence of catchments on coastal water quality. In Taiwan, traditional classroom learning has been used with regards to coastal revitalisation [63]. New York University, United States, has used such methods for learning about coastal urbanisation and environmental change [64].

### E-learning and blended learning

E-learning over small or large distances is by no means a new method. However, the recent covid-19 pandemic brought about new, albeit involuntary, opportunities for and experience from e-learning, since physical meetings became restricted in many parts of the world [65–67]. Thangajesu Satsish et al. [6] and Maatuk et al. [67] found that this forced online experiment created complementary methods to traditional classroom learning, and that a combination of the two could improve effectiveness, efficiency and interaction in learning, thereby encouraging active learner participation as promoted by the constructive alignment concept. Online learning necessitates more flexibility, support structures and instructional design and could require a greater extent and requirements regarding the organisation of learning activities [6, 61]. Unstable internet connections can be an obstacle in many countries, particularly at the low end of the income scale [65, 67]. According to some studies, e-learning tends to be less popular among university students than traditional classroom learning [5, 68], although opposing results have also been found [69]. E-learning is easily combined with online student interaction and enables students to join courses at sites far from where they reside, and such courses may attract large number of students. For instance, the LSI related university course “Sustainability perspectives on contemporary fisheries: Where have all the fishes gone?” [70] in Sweden (Swedish University of Agricultural Sciences) has e-learning students from all over Europe and other parts of the world who opt not to study in Sweden. The University of Mauritius has used an online platform for teaching coastal zone management, allowing students to participate from remote areas [71]. Many universities worldwide, such as Harvard University in the United States, École Polytechnique in France, and Hong Kong University of Science and Technology in China, offer fee-free online courses on a wide range of subjects including those related to the LSI.

The term blended learning has been defined as “the mixture of e-learning and classroom learning”, and uses the benefits of both methods [72]. A meta-analysis by Vo et al. [73] showed positive effects from blended learning on student performance, particularly in science, technology, engineering, and mathematics (STEM) fields. E-learning and blended learning may be a particular challenge, but still possible, in low-income countries [74]. Examples relating to the LSI include the State University of Malang in Indonesia, which has applied blended learning in the studies of beach sports [75]. The University of Aberdeen, United Kingdom, developed computer models for blended learning with the intention to improve three-dimensional understanding of coastal geological processes [76].

### Flipped classroom

Flipped (or inverted) classroom techniques have developed and increased in use over the past decade, particularly in the United States [77, 78]. The idea with the flipped classroom is, first, for the learner to study lectures, videos, blog posts, podcasts and similar material using computers or other technical devices at home before class. Such activities are, second, combined with meeting peers and lecturers at the university for learner-centered group discussions, problem solving and other activities relating to what has been done at home [77, 78]. This method is highly compatible with constructive alignment since participatory learner roles are encouraged. There has also been criticism against flipped classroom techniques for, e.g., being too technology dependent, for not clearly improving study results, and for having different effects on different learner groups [79]. Evidence on the overall effect of flipped classroom on learning performance appears to be quite ambiguous and case dependent [80]. Nevertheless, several student satisfaction studies have shown that university students tend to prefer flipped classroom to traditional teaching in higher education [81–84]. Li et al. [85] described the use of computer based methods at home in a landscape architecture course, where problems that arose with landscape design were discussed in class. There is also plenty of free online material available to additionally advise and facilitate flipped classroom teaching and learning about the LSI. For instance, the course module “Ocean Physics & Climate Change” in the United Kingdom (UCL, London) lets learners summarise the previous lecture using any chosen kind of technical support, as an initiating attempt to apply flipped classroom techniques [86].

### Problem-based learning

Problem-based learning (PBL) has been popular in higher education for several decades. Learning is performed in a group, which is presented with an open-ended problem. Problem-based learning is thus learner centered (as suggested in the constructive alignment paradigm) and develops group cooperation skills, communication skills, project management skills and real-world experiences [87]. Effects on critical thinking skills have been disputed, but a recent meta-analysis by Liu and Pásztor [88] found strong positive effects. Effects of PBL on learning effectiveness have been investigated with mixed outcomes, although effects on long-term learning tend to be positive compared to conventional classroom learning [89]. There is, however, still a great need to develop measurement instruments for properly evaluating PBL compared to other teaching methods [90]. In Taiwan, PBL has been used in higher education to study the revitalisation of a coastal area [63]. In the United States, PBL has been applied in higher education to study land subsidence and rising sea levels [91].

### Crossover learning

Crossover learning is an umbrella term for methods which bring together informal and formal learning. This could mean supervised visits and active participation at a museum, a conference hall, a stakeholder workshop, a poster session or virtually anything outside of the university walls [94–97]. Azmi et al. [92] found high (mostly > 4 on a scale from 1 to 5) student ratings for a teacher facilitated but student-led crossover learning event. Couper et al. [97] organised an event intended for crossover learning in Australia about turtle ecology and plastic pollution in the LSI. Active learner participation makes this method suitable in the framework of constructive alignment.

### Field trips and excursions

Field trips and excursions are costly but popular parts of courses that stimulate interest in the topic and provide first-hand experience, personal development and opportunities for social interaction. The learner gets to travel to a place where the subject taught is performed in practice, and the field trip or excursion is often combined with practical exercises [98]. Field trips and excursions enable unique kinds of learning that is not possible to replicate in class and are performed in a wide variety of fields, including natural and social sciences [99]. Activities often require substantial learner engagement, and thus conform well to constructive alignment. The University of South Florida, USA, arranges physical field trips in higher education focusing on coastal ecology, coastal geology, watersheds and coastal conservation [100]. An increasingly popular variety of field trips, which may be less costly than physical field trips, but offering less social interaction, is virtual field trips, where learners get to experience topics via computer. Virtual field trips with a partial coastal focus have been developed in earth and environmental sciences at Ankara University, Turkey [101]. A collaboration between several European universities offers virtual field trips on coastal geomorphology, sea-level changes, coastal adaptation to climate change, and socio-economic development focusing on a coastal bay [102].

### Game-based learning

Yet another learning field, which has gained popularity in recent decades, is game-based learning (GBL). A game is typically designed by experts and programmers, and played by the learner, who acquires knowledge and skills while playing actively, and have access to support from an instructor. According to Subhash et al. [103], Pellas and Mystakidis [104] and Zou et al. [105], GBL improves learner motivation and performance in higher education. However, gamification requires reliable and costly computer hardware and software programming, which must often be maintained and updated regularly [103]. GBL in higher education has been described in numerous studies, some of which have a certain relevance to sustainability and the LSI. For instance, Weines [106] evaluated GBL with focus on marine resource management. Keogh et al. [107] found that GBL regarding sports biomechanics increased learner engagement. Improved learning among university students was found by Türkistanli and Kuleyin [108] regarding a game-based decision support system in maritime transportation engineering. GBL can also improve learning of sustainability issues and conflict management according to Makri [109].

### Spacing and self-testing

Spacing (sometimes referred to as “spaced learning”) is a method developed by neuroscience, cognitive research and adjacent disciplines and aims at optimal training of the long-term memory. Highly intensive and compressed learning segments are repeated several times each and are interrupted by non-stimulus breaks [110]. Self-testing is performed by the learners themselves and involves testing their understanding of a topic by assessing the accuracy of memorised information [111]. These two methods can be very effective compared to other forms of learning, and this relative effectiveness is poorly known by both teachers and learners [110–112]. Searches in the databases Google Scholar and Web of Science indicated that self-testing is used in a wide range of scientific fields, and much more so than spacing. Both methods should nevertheless have high potential in LSI and sustainability teaching and learning.

### Continuous assessment

Continuous assessment in higher education has been defined as “the use of tests over a learning unit, and the accumulation of results in a final grade” [113]. Assessment may, for instance, be performed on a weekly basis [114]. Frequent assessment occasions could require more effort from the teacher, and may be difficult to finance in courses with low student numbers. Nevertheless, continuous assessment has overall been found to increase student engagement and improve learning outcomes [114]. Passing rates increased sharply among coastal engineering students at the University of Cadíz, Spain, after continuous assessment was introduced [115]. Jonas [33] developed a curriculum framework for a coastal and marine tourism education programme at Nelson Mandela University in South Africa and found that continuous assessment of students was particularly popular among stakeholders in the tourism sector.

### Formative assessment

According to Boston [116], “[a]ssessments become formative when the information is used to adapt teaching and learning to meet student needs”. Formative assessment can be continuous, but can also be very occasional [117]. Three practical examples are quizzing, peer feedback and teacher feedback [117]. However, the impact of formative assessment on student performance and satisfaction in higher education has only been subject to limited investigation, and the scant evidence available tends to be mixed [118]. There seems to be limited use of formative assessment in LSI teaching and learning in higher education.

## Discussion and conclusions

According to the overview presented in this paper, there is intensive development of new teaching methods ongoing with relevance for sustainability in the LSI. New methods tend to have a strong learner-centered focus, which is in line with the influential paradigm of constructive alignment. The pedagogy varies between courses and programmes and there is probably no “one size fits all” teaching and learning model for all LSI related courses. It is probably wise to maintain a high diversity in methods and select different methods for different purposes. It is likely that traditional classroom learning [5, 58] will continue to play a major role for teaching and learning sustainability in the LSI, preferably with a substantial role of active learner involvement. Experiences from the recent pandemic has highlighted the advantages and disadvantages with e-learning and blended learning [65–67] and that experience should be taken into account when developing or modifying LSI courses. E-learning and blended learning may attract additional students from far away in relation to where the course is taught. Flipped classroom techniques [77, 78] and problem-based learning [87, 88] may be suitable for some LSI related modules, courses and programmes, but less so for others.

Field trips and excursions [98, 99] are popular elements of various LSI courses and may improve learner satisfaction, increase student enrollment, and offer unique opportunities for learning, which may not always be available in classrooms or lecture halls. Game-based learning [103–105] may elevate motivation and bring variation to teaching and learning in an LSI setting, although it requires efforts to create and update games in order for them to be relevant.

Crossover learning [92–96] can probably be arranged as a part of a course, in order to attain variation, to increase student engagement and relate other activities to the real world in which the students will eventually work. Spacing and self-testing [110–112] have great potential for substantially improving learning efficiency, and we encourage teachers to explore these methods and possibly incorporate them in their curricula. Continuous assessment requires extra effort and resources, but may nevertheless improve passing rates of students [115]. The efficiency of formative assessment has yet to be investigated properly [118], but that may be a method worth exploring and evaluating in teaching and learning related to sustainability in the LSI. There are many additional novel teaching and learning methods available [59, 94], which were not explored in this study for reasons of brevity.

The current teaching and learning methods on sustainability in the LSI should also consider exposing the students to the policy interface between science and management; and stakeholder interactions. In this respect, the Stanford Center for Ocean Solutions and the Stanford Law School, at Stanford University, developed a course on: ‘The Outlaw Ocean Policy Practicum’. The course outcome resulted in two comprehensive student reports [119, 120], two extremely valuable and timely contributions to ocean and global sustainability, given the multitude of national and global ambitions and agreements to promote and strive for these wider goals. Two other aspects that need to be considered in relation to sustainability and equity are: (a) the inclusion of local and indigenous knowledge systems, to integrate cultural, social interactions and different worlds views, as part of a complex knowledge network that is important to inform decision-making processes [121], and the UNESCO initiative on ‘Local and Indigenous Knowledge System (LINKS)’: https://en.unesco.org/links; and (b) the inclusion of Ocean Literacy as a framework for enhancing knowledge sharing across all sectors of society and improving our understanding of the role that the oceans have in our well-being (UNESCO Ocean Literacy: https://ioc.unesco.org/topics/ocean-literacy and [122]).

To conclude, this paper has provided and discussed an overview of selected teaching and learning methods, which may be globally relevant in the context of sustainability in the LSI. Some of the newer methods have been explored more thoroughly in relation to education in other scientific disciplines, and we think it is time to consider them in teaching and learning about sustainability in the LSI as well. While there is a substantial variety in pedagogy and methods in sustainability education regarding the LSI, we hope that this paper can serve as a fact-base and inspiration to course leaders and programme developers in this area.

## References

[1] Clarke M (1971). Higher education in the Ancient World.

[2] Pedersen O (1997). The first universities: Studium generale and the origins of university education in Europe.

[3] Choudary SK (2008). Higher education in India: a socio-historical journey from ancient period to 2006–2007. J Educ Enquiry.

[4] Gu J, Li X, Wang L (2018). Higher education in China.

[5] Allen M, Bourhis J, Burrell N, Mabry E (2002). Comparing student satisfaction with distance education to traditional classrooms in higher education: a meta-analysis. Am J Distance Educ.

[6] Thangajesu Satsish M, Sornaganesh V, Sudha G, Chellama AV (2020). A study on shift of traditional classroom methods to online teaching methods in higher education. Int J Multidiscip Res Dev.

[7] Awacorach J, Jensen I, Lassen I, Olanya DR, Zakaria HL, Tabo GO (2021). Exploring transition in higher education: engagement and challenges in moving from teacher-centered to student-centered learning. J Problem Based Learn High Educ.

[8] Henson KT (2003). Foundations for learner-centered education: a knowledge base. Education.

[9] Kennedy D (2006). Writing and using learning outcomes: a practical guide.

[10] Biggs J (1996). Enhancing teaching through constructive alignment. High Educ.

[11] Biggs JB, Tang C (2011). Teaching for quality learning at university.

[12] McCann M (2016). Constructive alignment in economics teaching: a reflection on effective implementation. Teach High Educ.

[13] Hailikari T, Virtanen V, Vesalainen M, Postareff L (2022). Student perspectives on how different elements of constructive alignment support active learning. Act Learn High Educ.

[14] Loughlin C, Lygo-Baker S, Lindberg-Sand Ã (2021). Reclaiming constructive alignment. Eur J High Educ.

[15] Allan J (2006). Learning outcomes in higher education. Stud High Educ.

[16] Elken M, Tellmann SM (2019). Linking higher education and the world of work: learning outcomes and intermediary organisations. J Educ Work.

[17] Darawong C, Widayati A. Improving student satisfaction and learning outcomes with service quality of online courses: evidence from Thai and Indonesian higher education institutions. J Appl Res Higher Educ 2022. in press (online version available).

[18] Caspersen J, Frølich N, Muller J (2017). Higher education learning outcomes – ambiguity and change in higher education. Eur J Educ.

[19] Mintz K, Tal T (2018). The place of content and pedagogy in shaping sustainability learning outcomes in higher education. Environ Educ Res.

[20] Adam S (2008). Learning outcomes current developments in Europe: update on the issues and applications of learning outcomes associated with the Bologna process.

[21] Chemi T, Krogh L (2017). Co-creation in higher education.

[22] Dollinger M, Lodge J, Coates H (2018). Co-creation in higher education: towards a conceptual model. J Mark High Educ.

[23] Bovill C (2020). Co-creation in learning and teaching: the case for a whole-class approach in higher education. High Educ.

[24] Talley DM, North EW, Juhl AR, Timothy DA, Conde D, deBrouwer JF, Brown CA, Campbell LM, Garstecki T, Hall CJ, Meysman FJ, Nemerson DM, Souza Filho PW, Wood RJ (2003). Research challenges at the land–sea interface. Estuar Coast Shelf Sci.

[25] Pittman J, Armitage D (2016). Governance across the land–sea interface: a systematic review. Environ Sci Policy.

[26] Singh GG, Cottrell RS, Eddy TD, Cisneros-Montemayor AM (2021). Governing the land–sea interface to achieve sustainable coastal development. Front Mar Sci.

[27] Li D, Gan J, Hui R, Liu Z, Yu L, Lu Z, Dai M (2020). Vortex and biochemical dynamics for the hypoxia formation within the coastal transition zone off the Pearl River estuary. J Geophys Res Oceans.

[28] Kim JY, Naboa EE, Amidon F, Reeves MK, Miller SE. Hawaiian Islands coastal ecosystems: past, present, and future. Encyclopedia of the World’s Biomes; 2020. p. 157–74.

[29] Heckwolf MJ, Peterson A, Jänes H, Horne P, Künne J, Liversage K, Sajeva M, Reusch TBH, Kotta J (2021). From ecosystems to socio-economic benefits: a systematic review of coastal ecosystem services in the Baltic Sea. Sci Total Environ.

[30] Bryhn AC, Kraufvelin P, Bergström U, Vretborn M, Bergström L (2020). A model for disentangling dependencies and impacts among human activities and marine ecosystem services. Environ Manage.

[31] Kelly C (2016). ’I need the Sea and the Sea needs me’: symbiotic coastal policy narratives for human wellbeing and sustainability in the UK. Mar Policy.

[32] UN (2007). Percentage of total population living in coastal areas.

[33] Jonas L. A curriculum framework for undergraduate coastal and marine tourism university programmes. PhD thesis. Nelson Mandela University, Port Elizabeth; 2019.

[34] Wang H-C (2021). Adaptation of undesirable-output DEA for navigation safety in Taiwan international ports. Cogent Eng.

[35] Gacutan J, Galparsoro I, Pinarbasi K, Murillas A, Adewumi IJ, Praphotjanaporn T, Johnston EL, Findlay KP, Milligan BM (2022). Marine spatial planning and ocean accounting: synergistic tools enhancing integration in ocean governance. Mar Policy.

[36] Belgrano A, Novaglio C, Svedäng H, Villasante S, Melián CJ, Blenckner T, Bergström U, Bryhn A, Bergström L, Bartolino V, Sköld M, Tomczak M, Wikström SA, Hansen AS, Linke S, Emmerson R, Morf A, Tönnesson K (2021). Mapping and evaluating marine protected areas and ecosystem services: a transdisciplinary Delphi forecasting process framework. Front Ecol Evol.

[37] Brundiers K, Barth M, Cebrián G, Cohen M, Diaz L, Doucette-Remington S, Dripps W, Habron G, Harré N, Jarchow M, Losch K, Michel J, Mochizuki Y, Rieckmann M, Parnell R, Walker P, Zint M (2021). Key competencies in sustainability in higher education—toward an agreed-upon reference framework. Sustain Sci.

[38] Menon S, Suresh M (2020). Synergizing education, research, campus operations, and community engagements towards sustainability in higher education: a literature review. Int J Sustain High Educ.

[39] Martins AA, Mata TM, Costa CAV (2006). Education for sustainability: challenges and trends. Clean Technol Environ Policy.

[40] Leal Filho W, Raath S, Lazzarini B, Vargas VR, de Souza L, Anholon R, Quelhas OLG, Haddad R, Klavins M, Orlovic VL (2018). The role of transformation in learning and education for sustainability. J Clean Prod.

[41] Dunlop L, Rushton EAC (2022). Education for environmental sustainability and the emotions: implications for educational practice. Sustainability.

[42] Ocean Governance. COST OceanGov—Land−Sea Interactions Training School in Constanta. 2019. https://www.earthsystemgovernance.net/oceans/?p = 739 . Accessed 15 Nov 2022.

[43] City University of London. Maritime Operations and Management MSc. 2022. https://www.city.ac.uk/prospective-students/courses/postgraduate/maritime-operations-and-management. Accessed 15 Nov 2022.

[44] SLU. Climate change – effects on the landscape and potential solutions. 2022. https://www.slu.se/en/education/programmes-courses/course/LK0401/30003.2223/Climate-Change-Effects-on-the-Landscape-and-Potential-Solutions/. Accessed 15 Nov 2022.

[45] National Institute of Disaster Management. Coastal hazards management. 2021. https://www.youtube.com/watch?v = dFa3-DgfsE. Accessed 15 Nov 2022.

[46] HEC (2016). Curriculum of marine science.

[47] IOI Ocean Academy. IOI Thailand. 2019. https://www.ioinst.org/training/ioi-training-programmes-portfolio/ioi-thailand/. Accessed 15 Nov 2022.

[48] Hokkaido University. Course in human and ecological systems. 2022. https://www.ees.hokudai.ac.jp/kigaku/?page_id=53amp;lang=en. Accessed 15 Nov 2022.

[49] CLIVAR. CLIVAR-FIO Summer School on Ocean Macroturbulence and Its Role in Earth’s Climate. 2022. https://www.clivar.org/events/clivar-fio-summer-school-ocean-macroturbulence-and-its-role-earth%E2%80%99s-climate. Accessed 15 Nov 2022.

[50] OceanExpert. Marine GIS applications for coastal zone management. 2019. https://oceanexpert.org/event/2282. Accessed 15 Nov 2022.

[51] The University of Newcastle. Coastal and marine science. 2022. https://www.newcastle.edu.au/degrees/bachelor-of-coastal-and-marine-science. Accessed 15 Nov 2022.

[52] CMAUCH. Marine archaeology and underwater cultural heritage. 2022. http://www.cmauch.org/academics/. Accessed 15 Nov 2022.

[53] University of Florida. Sea level rise and coastal cities: science, policy and practice. 2022. https://www.law.ufl.edu/courses/spring-break-field-course-sea-level-rise-coastal-cities-science-policy-practice. Accessed 15 Nov 2022.

[54] McGill. Society, environment and sustainability. 2022. https://www.mcgill.ca/study/2022-2023/courses/envr-201. Accessed 15 Nov 2022.

[55] UC. Trabajo experimental en ecología marina (Experimental work in marine ecology). 2022. https://pregrado.bio.uc.cl/cursos/bio298m-trabajo-experimental-en-ecologia-marina/. Accessed 17 Nov 2022. (in Spanish)

[56] Scouller K (1998). The influence of assessment method on students’ learning approaches: multiple choice question examination versus assignment essay. High Educ.

[57] Pereira D, Assunção Flores M, Niklasson L (2016). Assessment revisited: a review of research in *Assessment and evaluation in Higher Education*. Assess Evaluation High Educ.

[58] Young S, Duncan HE (2014). Online and face-to-face teaching: how do student ratings --differ?. MERLOT J Online Learn Teach.

[59] Sinecen M (2018). Trends in e-learning.

[60] Castro MDB, Tumibay GM (2021). A literature review: efficacy of online learning courses for higher education institution using meta-analysis. Educ Inform Technol.

[61] Dolasinski MJ, Reynolds J. Microlearning in the higher education hospitality classroom. J Hosp Tour Educ 2022. (in press, electronic version available).

[62] Lasrado F, Kaul N (2020). Designing a curriculum in light of constructive alignment: a case study analysis. J Educ Bus.

[63] Ting K-H, Cheng C-T, Ting H-Y (2021). Introducing the problem/project-based learning as a learning strategy in University Social responsibility program – a study of local revitalization of Coastal Area, Yong-An District of Kaohsiung City. Mar Policy.

[64] Burt JA, Killilea ME, Ciprut S (2019). Coastal urbanization and environmental change: opportunities for collaborative education across a global network university. Reg Stud Mar Sci.

[65] Aboagye E, Yawson JA, Appiah KN (2020). COVID-19 and e-learning: the challenges of students in tertiary institutions. Social Educ Res.

[66] Amarneh BM, Alshurideh MT, Kurdi A, Obeidat BHZ. The impact of COVID-19 on e-learning: advantages and challenges. In: Proceedings of the international conference on artificial intelligence and computer vision (AICV 2021). Springer, Berlin; 2021. p. 75–89.

[67] Maatuk AM, Elberkawi EK, Aljawarneh S, Rashaideh H, Alharbi H (2022). The COVID-19 pandemic and E-learning: challenges and opportunities from the perspective of students and instructors. J Comput High Educ.

[68] Ashwin P, editor. Changing higher education. The development of learning and teaching. London: Routledge; 2005.

[69] Shahzad A, Hassan R, Aremu AY, Hussain A, Lodhi RN (2021). Effects of COVID-19 in E-learning on higher education institution students: the group comparison between male and female. Qual Quant.

[70] SLU. Sustainability perspectives on contemporary fisheries: Where have all the fishes gone? 2022. https://www.slu.se/en/departments/aquatic-resources1/education/the-course-sustainability-perspectives-on-contemporary-fisheries.-where-have-all-the-fishes-gone/#:~:text=%E2%80%9CWhere%20have%20all%20the%20fishes,future%20challenges%20for%20sustainable%20fisheries. Accessed 15 Nov 2022.

[71] Ramessur RT, Santally MI (2007). Computer mediated communication for effective teaching-learning of coastal zone management module. Int J Educ Dev Inf Commun Technol.

[72] Masie E, Bonk CJ (2006). The blended learning imperative. The handbook of blended learning: global perspectives, local designs.

[73] Vo HM, Zhu C, Diep NA (2017). The effect of blended learning on student performance at course-level in higher education: a meta-analysis. Stud Educ Eval.

[74] Khan MEI (2021). Deploying blended learning in the new normal pedagogy. challenges and prospects in Bangladesh. Int J Asian Educ.

[75] Roesdiyanto M, Sulistyorini M, Fadhli NR, Taufik M. The use of blended learning model integrated with learning management system in beach volleyball learning subject in Faculty of Sports Science, State University of Malang. In: Proceedings of the 2nd international conference on sports sciences and health 2018 (2nd ICSSH 2018). Atlantis Press, Paris; 2019. p. 156–159.

[76] Bond CE, Cawood AJ (2021). A role for virtual outcrop models in blended learning – improved 3D thinking and positive perceptions of learning. Geoscience Commun.

[77] O’Flaherty J, Phillips C, Karanicolas S, Snelling C, Winning T (2015). The use of flipped classrooms in higher education: a scoping review. Internet High Educ.

[78] Lundin M, Bergviken Rensfeldt A, Hillman T, Lantz-Andersson A, Peterson L (2018). Higher education dominance and siloed knowledge: a systematic review of flipped classroom research. Int J Educ Technol High Educ.

[79] He W, Holton A, Gu H, Warschauer M, Farkas G (2019). Differentiated impact of flipped instruction: when would flipped instruction work or falter?. Int J Teach Learn High Educ.

[80] Jung H, Park SW, Kim HS, Park J (2022). The effects of the regulated learning-supported flipped classroom on student performance. J Comput High Educ.

[81] Comber DPM, Brady-Van den Bos M (2018). Too much, too soon? A critical investigation into factors that make flipped classrooms effective. High Educ Res Dev.

[82] Kraut AS, Omron R, Caretta-Weyer H, Jordan J, Manthey D, Wolf SJ, Yarris LM, Johnson S, Kornegay J (2019). The flipped classroom: a critical appraisal. West J Emerg Med.

[83] Polat H, Karabatak S (2022). Effect of flipped classroom model on academic achievement, academic satisfaction and general belongingness. Learn Environ Res.

[84] Lin G-Y, Wang Y-S, Lee YN (2022). Investigating factors affecting learning satisfaction and perceived learning in flipped classrooms: the mediating effect of interaction. Interact Learn Environ.

[85] Li D-H, Jiang B-S, Li H-Y, Liu X-P (2021). Design of experiment course “Computer-aided landscape design” based on flipped classroom. Comput Appl Eng Educ.

[86] Tsamado M. Flipped classroom ideas for ocean physics and climate change module #GEOL0022. 2022. https://www.ucl.ac.uk/earth-sciences/news/2021/dec/flipped-classroom-ideas-ocean-physics-climate-change-module-geol0022. Accessed 03 Jun 2022.

[87] Nilson LB (2010). Teaching at its best: a research-based resource for college instructors.

[88] Liu Y, Pásztor A (2022). Effects of problem-based learning instructional intervention on critical thinking in higher education: a meta-analysis. Think Skills Creativity.

[89] Yew EHJ, Goh K (2016). Problem-based learning: an overview of its process and impact on learning. Health Prof Educ.

[90] Guo P, Saab N, Post LS, Admiraal W (2020). A review of project-based learning in higher education: student outcomes and measures. Int J Educ Res.

[91] Tewari S. Problem-based learning as a pedagogy for individual students - quantifying the long-term effects of land subsidence and rising sea levels in coastal areas for greater student engagement. In: Proceedings of the 126th ASEE annual conference and exposition (2019, Tampa, FL), American Society for Engineering Education (ASEE), Jun 2019. ASEE, Tampa; 2021.

[92] Azmi N, Yaakob NS, Jasamai M, Chua EW (2018). Crossover learning through a health campaign integrated in a bachelor of Pharmacy curriculum. ASEAN J Teach Learn High Educ.

[93] Kalyani D, Rajasekaran K (2018). Innovative teaching and learning. J Appl Adv Res.

[94] Sharples M (2018). Practical pedagogy.

[95] Srinivasa KG, Kurni M, Saritha K (2022). Learning, teaching, and assessment methods for contemporary learners. Pedagogy for the digital generation.

[96] Johnsi Priya J. Crossover learning for formal and informal learning. In: Lawrence A, A.S, editor. Emerging trends of psycho-technological approaches in heutagogy. Tamiil Nadu Open University, Chennai; 2022. p. 121–123.

[97] Couper P, Go M, Amey A, Limpus C (2019). The world science festival Brisbane’s loggerhead turtle hatchery: a case study. Teach Sci.

[98] Larsen C, Walsh C, Almond N, Myers C (2017). The “real value” of field trips in the early weeks of higher education: the student perspective. Educ Stud.

[99] Fedesco HN, Cavin D, Henares R (2020). Field-based learning in higher education: exploring the benefits and possibilities. J Scholarsh Teach Learn.

[100] USF. Coastal field trips. 2022. https://www.usf.edu/marine-science/community-engagement/coastal-field-trips.aspx. Accessed 23 Jun 2022.

[101] Çaliskan O (2011). Virtual field trips in education of earth and environmental sciences. Procedia Soc Behav Sci.

[102] CIVIS. Virtual field trips on geomorphology and quaternary geology. 2022. https://civis.eu/en/civis-courses/virtual-field-trips-on-geomorphology-and-quaternary-geology Accessed 23 Jun 2022.

[103] Subhash S, Cudney EA (2018). Gamified learning in higher education: a systematic review of the literature. Comput Hum Behav.

[104] Pellas N, Mystakidis S (2020). A systematic review of research about game-based learning in virtual worlds. J Univers Comput Sci.

[105] Zou D, Zhang R, Xie H, Wang FL (2021). Digital game-based learning of information literacy: effects of gameplay modes on university students’ learning performance, motivation, self-efficacy and flow experiences. Australasian J Educational Technol.

[106] Weines J. Game-based learning for marine resource management: reflections on using games in the Bachelor of Science in Fisheries and Aquaculture. PhD thesis. UiT The Arctic University of Norway, Troms&#248. 2021.

[107] Keogh JWL, Moro C, Knudson D. Promoting learning of biomechanical concepts with game-based activities. Sports Biomech 2022. in press. 10.1080/14763141.2020.1845470 33874853

[108] Türkistanli TT, Kuleyin B (2022). Game-based learning for better decision-making: a collision prevention training for maritime transportation engineering students. Comput Appl Eng Educ.

[109] Makri E (2021). Can game-based learning facilitate civics, negotiation and conflict management attributes? Research evidence from greek university students. Int J Cross-Discip Subj Educ.

[110] Kelley P, Whatson T (2013). Making long-term memories in minutes: a spaced learning pattern from memory research in education. Front Hum Neurosci.

[111] Rodriguez F, Fisher C, Zhou N, Warschauer M, Massimelli Sewall J (2021). Student spacing and self-testing strategies and their associations with learning in an upper division microbiology course. SN Soc Sci.

[112] Schwerter J, Dimpfl T, Bleher J, Murayama K (2022). Benefits of additional online practice opportunities in higher education. Internet High Educ.

[113] Miller A, Imrie B, Cox K (1998). Student assessment in higher education: a handbook for assessing performance.

[114] Holmes N (2018). Engaging with assessment: increasing student engagement through continuous assessment. Act Learn High Educ.

[115] Navarro-Pons M, Moreno L, Muñoz Pérez JJ, Anfuso Melfi G, Román-Sierra J. Success on increasing number of students that pass the coastal engineering subject. In: EDULEARN14 proceedings 6th international conference on education and new learning technologies Barcelona. 2014. p. 4443–4448.

[116] Boston C (2002). The concept of formative assessment. Prac Assess Res Eval.

[117] Yorke M (2003). Formative assessment in higher education: moves towards theory and the enhancement of pedagogic practice. High Educ.

[118] Morris R, Perry T, Wardle L (2021). Formative assessment and feedback for learning in higher education: a systematic review. Rev Educ.

[119] Stanford Law & Policy Practicum. The Outlaw Ocean: an exploration of policy solutions to address illegal fishing and forced labor in the seafood industry. Stanford: Stanford Center for Ocean Solutions and the Stanford Law School; 2020.

[120] Stanford Law & Policy Practicum (2021). The Outlaw Ocean: business and technology solutions that address illegal fishing and labor abuses in Seafood Supply Chains.

[121] UNESCO. Report of the UNESCO expert meeting on indigenous knowledge and climate change in Africa, Nairobi, Kenya, 27–28 June 2018, UNESCO; 2020. p. 52.

[122] NOAA (2020). Ocean literacy: the essential principles and fundamental concepts of ocean sciences for learners of all ages.

